# (*E*)-3-(4-Chloro­phen­yl)-1-(2-thien­yl)prop-2-en-1-one

**DOI:** 10.1107/S1600536808022782

**Published:** 2008-07-26

**Authors:** Hoong-Kun Fun, Samuel Robinson Jebas, P. S. Patil, S. M. Dharmaprakash

**Affiliations:** a X-ray Crystallography Unit, School of Physics, Universiti Sains Malaysia, 11800 USM, Penang, Malaysia; b Department of Studies in Physics, Mangalore University, Mangalagangotri, Mangalore 574 199, India

## Abstract

The title compound, C_13_H_9_ClOS, adopts an *E* configuration with respect to the C=C double bond of the propenone unit. The thienyl and benzene rings are slightly twisted from each other, making a dihedral angle of 6.38 (3)°. An intra­molecular C—H⋯O hydrogen bond generates an *S*(5) ring motif. A weak inter­molecular C—H⋯O inter­action, a short intra­molecular S⋯O contact [2.932 (2) Å] and two π–π inter­actions between the thienyl and benzene rings are observed. The centroid–centroid distances of the π–π inter­actions are 3.7899 (16) and 3.7891 (16) Å.

## Related literature

For related literature on chalcone derivatives, see: Agrinskaya *et al.* (1999 ▶); Gu, Ji, Patil & Dharmaprakash (2008 ▶); Gu, Ji, Patil, Dharmaprakash & Wang (2008 ▶); Fun *et al.* (2008 ▶); Patil *et al.* (2006 ▶); Patil, Dharmaprakash *et al.* (2007 ▶); Patil, Fun *et al.* (2007 ▶). For bond-length data, see: Allen *et al.* (1987 ▶). For graph-set analysis of hydrogen bonding, see: Bernstein *et al.* (1995 ▶).
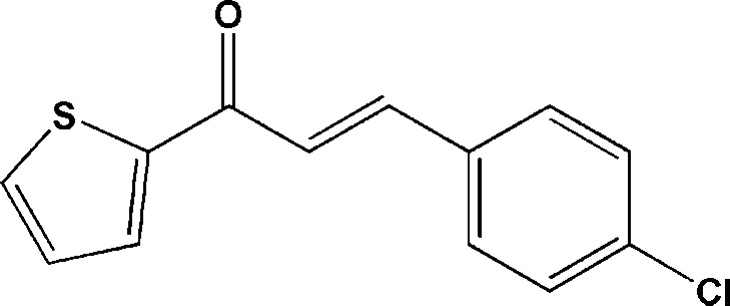


## Experimental

### 

#### Crystal data

C_13_H_9_ClOS
*M* *_r_* = 248.71
Monoclinic, 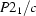

*a* = 5.7023 (3) Å
*b* = 13.3576 (8) Å
*c* = 14.7017 (10) Å
β = 96.735 (4)°
*V* = 1112.09 (12) Å^3^
*Z* = 4
Mo *K*α radiation
μ = 0.50 mm^−1^
*T* = 100.0 (1) K
0.45 × 0.13 × 0.12 mm

#### Data collection

Bruker SMART APEXII CCD area-detector diffractometer
Absorption correction: multi-scan (**SADABS**; Bruker, 2005 ▶) *T*_min_ = 0.804, *T*_max_ = 0.942
12436 measured reflections
3238 independent reflections
2546 reflections with *I* > 2σ(*I*)
*R*_int_ = 0.030

#### Refinement

*R*[*F* ^2^ > 2σ(*F* ^2^)] = 0.068
*wR*(*F* ^2^) = 0.199
*S* = 1.08
3238 reflections
145 parameters
H-atom parameters constrained
Δρ_max_ = 1.76 e Å^−3^
Δρ_min_ = −0.49 e Å^−3^

### 

Data collection: *APEX2* (Bruker, 2005 ▶); cell refinement: *APEX2*; data reduction: *SAINT* (Bruker, 2005 ▶); program(s) used to solve structure: *SHELXTL* (Sheldrick, 2008 ▶); program(s) used to refine structure: *SHELXTL*; molecular graphics: *SHELXTL*; software used to prepare material for publication: *SHELXTL* and *PLATON* (Spek, 2003 ▶).

## Supplementary Material

Crystal structure: contains datablocks global, I. DOI: 10.1107/S1600536808022782/is2316sup1.cif

Structure factors: contains datablocks I. DOI: 10.1107/S1600536808022782/is2316Isup2.hkl

Additional supplementary materials:  crystallographic information; 3D view; checkCIF report

## Figures and Tables

**Table 1:** Hydrogen-bond geometry (Å, °)

*D*—H⋯*A*	*D*—H	H⋯*A*	*D*⋯*A*	*D*—H⋯*A*
C7—H7*A*⋯O1	0.93	2.50	2.825 (4)	101
C13—H13*A*⋯O1^i^	0.93	2.58	3.389 (4)	145

Symmetry code: (i) 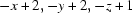.

**Fig. 1.**
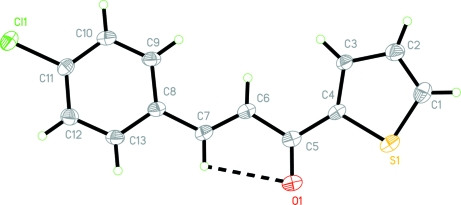
The molecular structure of the title compound, showing 50% probability displacement ellipsoids and the atom numbering scheme.

**Fig. 2.**
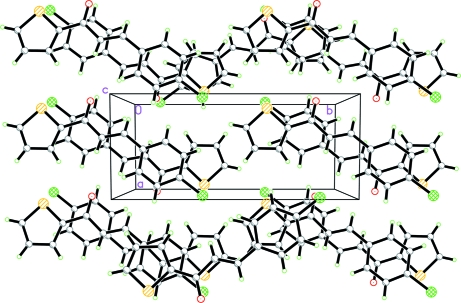
The crystal packing of the title compound, viewed along the c axis.

## supplementary crystallographic information

### Comment

Since some chalcone derivatives have shown to be potential nonlinear optical materials (Agrinskaya *et al.*, 1999), a series of new chalcone derivatives have been prepared in our laboratory (Gu, Ji, Patil & Dharmaprakash, 2008; Gu, Ji, Patil, Dharmaprakash & Wang, 2008; Fun *et al.*, 2008; Patil, Dharmaprakash *et al.*, 2007; Patil, Fun *et al.*, 2007; Patil *et al.*, 2006). As part of the ongoing investigation, the title compound has recently been prepared and its crystal structure is reported here.

In the crystal structure of the title compound, (I), the molecule exhibits an *E* configuration with respect to the C6═C7 double bond with the C5–C6–C7–C8 torsion angle being 180.0 (3)°. The bond lengths and bond angles in (I) are found to have normal values (Allen *et al.*, 1987). The thienyl (S1/C1—C4) and benzene (C8—C13) rings are essentially planar with the maximum deviation from planarity being -0.001 (2) Å for atom S1 and 0.011 (3) Å for atom C8. The thienyl ring and the benzene ring are slightly twisted from each other with a dihedral angle of 6.38 (8)°.

An intramolecular C—H···O hydrogen bond generates a ring motif S(5) (Bernstein *et al.*, 1995). The intramolecular short S···O contact [2.932 (2) Å] stabilizes the molecular conformation. The crystal packing is consolidated by a weak intermolecular C—H···O interaction. Two π–π interactions with the centroid-to-centroid distances of 3.7899 (16) and 3.7891 (16) Å are observed (symmetry codes: 1 - *x*, 1/2 + *y*, 1/2 - *z*; 1 - *x*, -1/2 + *y*, 1/2 - *z*).

### Experimental

The compound (I) was synthesized by the condensation of 4-chlorobenzaldehyde (0.01 mol, 1.49 mg) with 2-acetylthiophene (0.01 mol, 1.07 ml) in methanol (60 ml) in the presence of a catalytic amount of sodium hydroxide solution (5 ml, 30%). After stirring (10 h), the contents of the flask were poured into ice-cold water (500 ml) and left to stand for 5 h. The resulting crude solid was filtered and dried. The precipitated compound was recrystallized from N, *N*-dimethylformamide (DMF).

### Refinement

H atoms were positioned geometrically (C—H = 0.93 Å) and refined using a riding model, with *U*_iso_(H) = 1.2*U*_eq_(C). The highest peak in the difference Fourier map is located 0.82 Å from atom S1.

### Crystal data

**Table:**

C_13_H_9_ClOS	*F*_000_ = 512
*M**_r_* = 248.71	*D*_x_ = 1.485 Mg m^−^^3^
Monoclinic, *P*2_1_/*c*	Mo *K*α radiation λ = 0.71073 Å
Hall symbol: -P 2ybc	Cell parameters from 3595 reflections
*a* = 5.7023 (3) Å	θ = 2.8–29.8º
*b* = 13.3576 (8) Å	µ = 0.50 mm^−^^1^
*c* = 14.7017 (10) Å	*T* = 100.0 (1) K
β = 96.735 (4)º	Needle, colourless
*V* = 1112.09 (12) Å^3^	0.45 × 0.13 × 0.12 mm
*Z* = 4	

### Data collection

**Table:**

Bruker SMART APEXII CCD area-detector diffractometer	3238 independent reflections
Radiation source: fine-focus sealed tube	2546 reflections with *I* > 2σ(*I*)
Monochromator: graphite	*R*_int_ = 0.030
*T* = 100.0(1) K	θ_max_ = 30.1º
φ and ω scans	θ_min_ = 2.8º
Absorption correction: multi-scan(*SADABS*; Bruker, 2005)	*h* = −8→8
*T*_min_ = 0.804, *T*_max_ = 0.942	*k* = −16→18
12436 measured reflections	*l* = −20→15

### Refinement

**Table:**

Refinement on *F*^2^	Secondary atom site location: difference Fourier map
Least-squares matrix: full	Hydrogen site location: inferred from neighbouring sites
*R*[*F*^2^ > 2σ(*F*^2^)] = 0.068	H-atom parameters constrained
*wR*(*F*^2^) = 0.199	*w* = 1/[σ^2^(*F*_o_^2^) + (0.0986*P*)^2^ + 1.8996*P*] where *P* = (*F*_o_^2^ + 2*F*_c_^2^)/3
*S* = 1.08	(Δ/σ)_max_ = 0.001
3238 reflections	Δρ_max_ = 1.76 e Å^−^^3^
145 parameters	Δρ_min_ = −0.49 e Å^−^^3^
Primary atom site location: structure-invariant direct methods	Extinction correction: none

### Special details

**Table:**

Experimental. The data was collected with the Oxford Cyrosystem Cobra low-temperature attachment.
Geometry. All e.s.d.'s (except the e.s.d. in the dihedral angle between two l.s. planes) are estimated using the full covariance matrix. The cell e.s.d.'s are taken into account individually in the estimation of e.s.d.'s in distances, angles and torsion angles; correlations between e.s.d.'s in cell parameters are only used when they are defined by crystal symmetry. An approximate (isotropic) treatment of cell e.s.d.'s is used for estimating e.s.d.'s involving l.s. planes.
Refinement. Refinement of *F*^2^ against ALL reflections. The weighted *R*-factor *wR* and goodness of fit *S* are based on *F*^2^, conventional *R*-factors *R* are based on *F*, with *F* set to zero for negative *F*^2^. The threshold expression of *F*^2^ > σ(*F*^2^) is used only for calculating *R*-factors(gt) *etc*. and is not relevant to the choice of reflections for refinement. *R*-factors based on *F*^2^ are statistically about twice as large as those based on *F*, and *R*- factors based on ALL data will be even larger.

### Fractional atomic coordinates and isotropic or equivalent isotropic displacement parameters (Å^2^)

**Table:**

	*x*	*y*	*z*	*U*_iso_*/*U*_eq_	
S1	0.90267 (12)	1.35871 (6)	0.43901 (5)	0.0277 (2)	
Cl1	0.00412 (13)	0.64087 (5)	0.29914 (5)	0.0299 (2)	
O1	0.9797 (4)	1.14167 (16)	0.44334 (16)	0.0327 (5)	
C2	0.4870 (5)	1.4144 (2)	0.3764 (2)	0.0293 (6)	
H2A	0.3575	1.4550	0.3579	0.035*	
C3	0.4820 (5)	1.3077 (2)	0.37026 (19)	0.0224 (5)	
H3A	0.3505	1.2703	0.3476	0.027*	
C4	0.7018 (5)	1.2675 (2)	0.40300 (18)	0.0223 (5)	
C5	0.7766 (5)	1.1625 (2)	0.4128 (2)	0.0250 (6)	
C6	0.5981 (5)	1.0853 (2)	0.3844 (2)	0.0268 (6)	
H6A	0.4539	1.1042	0.3529	0.032*	
C7	0.6405 (5)	0.9890 (2)	0.40334 (19)	0.0240 (5)	
H7A	0.7868	0.9732	0.4349	0.029*	
C8	0.4777 (5)	0.9050 (2)	0.37894 (18)	0.0222 (5)	
C9	0.2473 (5)	0.9190 (2)	0.3366 (2)	0.0248 (6)	
H9A	0.1911	0.9837	0.3252	0.030*	
C10	0.1021 (5)	0.8388 (2)	0.31147 (19)	0.0238 (5)	
H10A	−0.0501	0.8491	0.2827	0.029*	
C1	0.7028 (5)	1.4507 (2)	0.4124 (2)	0.0301 (6)	
H1A	0.7357	1.5184	0.4211	0.036*	
C12	0.4120 (5)	0.7255 (2)	0.3732 (2)	0.0259 (6)	
H12A	0.4659	0.6607	0.3856	0.031*	
C13	0.5557 (5)	0.8070 (2)	0.39794 (19)	0.0238 (5)	
H13A	0.7068	0.7965	0.4277	0.029*	
C11	0.1866 (5)	0.7422 (2)	0.32979 (19)	0.0224 (5)	

### Atomic displacement parameters (Å^2^)

**Table:**

	*U*^11^	*U*^22^	*U*^33^	*U*^12^	*U*^13^	*U*^23^
S1	0.0200 (3)	0.0344 (4)	0.0281 (4)	−0.0065 (3)	0.0004 (3)	−0.0013 (3)
Cl1	0.0258 (4)	0.0293 (4)	0.0341 (4)	−0.0077 (3)	0.0020 (3)	−0.0027 (3)
O1	0.0221 (10)	0.0339 (12)	0.0397 (13)	−0.0002 (8)	−0.0060 (9)	0.0006 (9)
C2	0.0217 (13)	0.0321 (15)	0.0342 (16)	0.0004 (11)	0.0034 (11)	−0.0023 (12)
C3	0.0171 (11)	0.0235 (12)	0.0262 (13)	−0.0011 (9)	0.0012 (9)	−0.0021 (10)
C4	0.0174 (11)	0.0281 (13)	0.0207 (12)	−0.0040 (10)	0.0001 (9)	−0.0028 (10)
C5	0.0201 (12)	0.0304 (14)	0.0243 (13)	−0.0007 (10)	0.0014 (10)	−0.0009 (10)
C6	0.0191 (12)	0.0287 (14)	0.0312 (15)	−0.0021 (10)	−0.0029 (10)	0.0005 (11)
C7	0.0172 (11)	0.0281 (13)	0.0262 (13)	−0.0028 (10)	0.0007 (9)	−0.0002 (10)
C8	0.0173 (11)	0.0266 (13)	0.0224 (12)	0.0009 (9)	0.0016 (9)	−0.0015 (10)
C9	0.0170 (12)	0.0288 (14)	0.0279 (14)	0.0025 (10)	−0.0003 (10)	0.0004 (11)
C10	0.0170 (11)	0.0307 (13)	0.0233 (13)	0.0012 (10)	0.0004 (9)	0.0023 (10)
C1	0.0288 (14)	0.0307 (14)	0.0318 (15)	−0.0076 (11)	0.0072 (11)	−0.0024 (12)
C12	0.0224 (13)	0.0252 (13)	0.0300 (14)	0.0007 (10)	0.0031 (10)	0.0038 (11)
C13	0.0171 (11)	0.0279 (13)	0.0263 (13)	0.0024 (10)	0.0018 (9)	0.0036 (10)
C11	0.0196 (12)	0.0256 (13)	0.0227 (13)	−0.0048 (10)	0.0050 (9)	0.0003 (10)

### Geometric parameters (Å, °)

**Table:**

S1—C1	1.691 (3)	C7—C8	1.473 (4)
S1—C4	1.713 (3)	C7—H7A	0.9300
Cl1—C11	1.735 (3)	C8—C9	1.398 (4)
O1—C5	1.224 (3)	C8—C13	1.400 (4)
C2—C1	1.369 (4)	C9—C10	1.377 (4)
C2—C3	1.428 (4)	C9—H9A	0.9300
C2—H2A	0.9300	C10—C11	1.393 (4)
C3—C4	1.397 (4)	C10—H10A	0.9300
C3—H3A	0.9300	C1—H1A	0.9300
C4—C5	1.468 (4)	C12—C11	1.385 (4)
C5—C6	1.474 (4)	C12—C13	1.386 (4)
C6—C7	1.333 (4)	C12—H12A	0.9300
C6—H6A	0.9300	C13—H13A	0.9300
			
C1—S1—C4	92.14 (14)	C9—C8—C7	122.6 (3)
C1—C2—C3	112.8 (3)	C13—C8—C7	119.1 (2)
C1—C2—H2A	123.6	C10—C9—C8	121.3 (3)
C3—C2—H2A	123.6	C10—C9—H9A	119.4
C4—C3—C2	110.6 (2)	C8—C9—H9A	119.4
C4—C3—H3A	124.7	C9—C10—C11	119.0 (2)
C2—C3—H3A	124.7	C9—C10—H10A	120.5
C3—C4—C5	129.8 (2)	C11—C10—H10A	120.5
C3—C4—S1	111.9 (2)	C2—C1—S1	112.5 (2)
C5—C4—S1	118.2 (2)	C2—C1—H1A	123.7
O1—C5—C4	120.3 (3)	S1—C1—H1A	123.7
O1—C5—C6	122.5 (3)	C11—C12—C13	118.9 (3)
C4—C5—C6	117.2 (2)	C11—C12—H12A	120.6
C7—C6—C5	120.9 (3)	C13—C12—H12A	120.6
C7—C6—H6A	119.5	C12—C13—C8	121.2 (2)
C5—C6—H6A	119.5	C12—C13—H13A	119.4
C6—C7—C8	126.2 (3)	C8—C13—H13A	119.4
C6—C7—H7A	116.9	C12—C11—C10	121.3 (2)
C8—C7—H7A	116.9	C12—C11—Cl1	119.3 (2)
C9—C8—C13	118.3 (3)	C10—C11—Cl1	119.3 (2)
			
C1—C2—C3—C4	0.0 (4)	C6—C7—C8—C13	175.3 (3)
C2—C3—C4—C5	177.7 (3)	C13—C8—C9—C10	−2.0 (4)
C2—C3—C4—S1	−0.2 (3)	C7—C8—C9—C10	177.5 (3)
C1—S1—C4—C3	0.2 (2)	C8—C9—C10—C11	0.8 (4)
C1—S1—C4—C5	−177.9 (2)	C3—C2—C1—S1	0.2 (3)
C3—C4—C5—O1	−180.0 (3)	C4—S1—C1—C2	−0.2 (3)
S1—C4—C5—O1	−2.3 (4)	C11—C12—C13—C8	−0.6 (4)
C3—C4—C5—C6	0.2 (5)	C9—C8—C13—C12	1.9 (4)
S1—C4—C5—C6	177.9 (2)	C7—C8—C13—C12	−177.6 (3)
O1—C5—C6—C7	10.0 (5)	C13—C12—C11—C10	−0.6 (4)
C4—C5—C6—C7	−170.1 (3)	C13—C12—C11—Cl1	−179.6 (2)
C5—C6—C7—C8	180.0 (3)	C9—C10—C11—C12	0.5 (4)
C6—C7—C8—C9	−4.1 (5)	C9—C10—C11—Cl1	179.5 (2)

### Hydrogen-bond geometry (Å, °)

**Table:**

*D*—H···*A*	*D*—H	H···*A*	*D*···*A*	*D*—H···*A*
C7—H7A···O1	0.93	2.50	2.825 (4)	101
C13—H13A···O1^i^	0.93	2.58	3.389 (4)	145

Symmetry codes: (i) −*x*+2, −*y*+2, −*z*+1.
